# Editorial: Impact of COVID-19 on healthcare professions education

**DOI:** 10.3389/fmed.2023.1265811

**Published:** 2023-08-07

**Authors:** Pradeep Kumar Sahu, Hakki Dalçik

**Affiliations:** ^1^Centre for Medical Sciences Education, Faculty of Medical Sciences, The University of the West Indies, St. Augustine, Trinidad and Tobago; ^2^School of Medicine, Istanbul Aydin University, Istanbul, Türkiye

**Keywords:** COVID-19, healthcare profession, digital learning, face-to-face teaching, life-long learning

The COVID-19 pandemic caused a major disruptive change in the educational system, including healthcare professions education. The highly contagious nature of the virus forced healthcare professions education to suspend or cancel face-to-face lectures, labs, and clinical teachings (1). While the pandemic will be remembered as a source of disruption, many will likely see it as a game-changer for the transformation of healthcare professions education. Due to the pandemic restriction and closure of educational institutions, educators and healthcare professionals shifted from real-time physical teaching to virtual learning with substantial changes in curriculum including the delivery and assessment modes (2).

In the post-pandemic era, face-to-face education has already been restored, but the integration of technology is highly likely to be an essential component of the future of health professions education. The present topic aimed to explore alternative teaching modes adopted in healthcare professions education due to the impact of the COVID-19 pandemic. We invited articles on various topics on scientific research and advances in innovations in teaching, learning, and evaluation in healthcare professions education. The published articles have highlighted the challenges as well as meaningful ideas and solutions to integrate technology in healthcare teaching and training.

Students and teachers had no choice but to adopt remote learning for the smooth transition of the curriculum. They came across numerous challenges such as poor technological infrastructure, lack of non-verbal communication, lack of clinical skills development, improper handling of virtual learning platforms, and lack of collaborative learning. Engaging students in the virtual learning process was also a herculean task for the instructors (Sarkar et al.; Hertling et al.). Gradually, students and teachers became acquainted with these new ways of knowledge transfer and remote learning. At the beginning of the virtual learning, students were enthusiastic and showed a positive attitude toward the new format of learning, whereas many teachers viewed it negatively because of the challenges to operate the digital platform. Subsequently, teachers were able to acquire technology skills and be comfortable with online teaching (Hertling et al.).

To continue with digital learning for the long term beyond COVID-19, it must be learner-friendly and diversified in terms of teaching and assessment. Teachers need to utilize both synchronous and asynchronous learning more efficiently. Yang et al. constructed a student-centered diversified online method to teach neurology by combining the advantages of modern information technology and scientific research. The teaching method consisted of diverse teaching resources (Online courses, MOOCs, virtual reality simulation systems), diverse teaching services (question-answer sessions, discussion forums, teachers' feedback), and diverse teaching processes (multifarious teaching and assessment methods). It promoted students' interest in the subject, and they mastered the teaching content. It allowed teachers to reform their current teaching practices and improve the quality of teaching during the pandemic and for future education.

Practice-based clinical learning is a critical component of medical and healthcare training. It was challenging for the faculty and clinical preceptors to create an effective alternative to clinical learning when real-time classes were suspended during the pandemic (Bawadi, Shami et al.). Considering the safety of the students, family members, and patients, switching to virtual clinical teaching was considered necessary. However, it did not satisfy many preceptors and students due to the limited exposure to a real-time clinical teaching. Some students favored a real-time clinical learning environment with necessary protective measures (Bawadi, Rahim et al.). On the other hand, there is also evidence of students' positive outcomes in learning clinical skills by integrating appropriate technology. For example, Wang L. et al. found in their study that a virtual simulation operation (VSO) contributes to high-quality engagement in clinical skill courses. Students who used a VSO performed better in the clinical examinations compared to those who did not. It indicates that the proper use of technology, more specifically the VSO, can improve students' clinical skills operation. The outcomes of these studies set the base for future research to design effective and competency-based curriculum for online clinical learning using proper technology.

The COVID-19 pandemic opened the way to innovations in teaching adopting advanced digital technology such as e-learning, virtual simulation, podcasts, Siilos, Blackboard Collaborate, and many more (Wang M.-C. et al.). Podcasts are become increasingly popular in healthcare professions education. Healthcare professions education in Western countries has already integrated podcasts into curriculum design and delivery. The instant message application “Siilo” is another digital technology which has grown in popularity among physicians and health professionals during the pandemic. Siilo can create multiple folders in the smartphones or laptops which can store text messages, pictures, videos, and documents of patients separately. It appears to be a promising tool for facilitating case-based learning in health professions education. However, there is limited evidence showing the effectiveness of these digital technologies as teaching tools. More rigorous studies evaluating the students' learning outcomes and behavioral changes need to be performed to prove the significance of podcasts and Siilo compared to traditional educational modalities (Shahar et al.).

Health professionals require continuous and life-long learning to improve their competency level in their respective areas. Integration of technology in continuing medical education (CME) has undergone perpetual and progressive changes during COVID-19. Healthcare professionals used cloud classrooms which provided them with greater learning and exchange opportunities by attending virtual meetings and academic discussions from their homes without traveling anywhere. A cloud classroom plays a vital role in promoting learning experiences and developing the competency level of health professionals through a series of seminars, meetings, case discussions, special lectures, and interactive sessions (Chen et al.).

Good communication skills are a key attribute of a healthcare service. Therefore, every health profession student should demonstrate effective communication and interpersonal skills to become a competent healthcare professional. Assessment of these skills has always been a difficult job for the teachers. During the pandemic, assessing students' communication skills via *telehealth* provided a useful opportunity with the growing use of online environments. Wright et al. found that non-verbal communication is more difficult to assess using *telehealth* compared to verbal communication. To use *telehealth* effectively, proper training is required for teachers responsible for assessing students' communication and interpersonal skills.

The pandemic not only caused disruption in healthcare professions education but also created the issue of poor access to healthcare facilities. Due to lockdowns and restriction policies, self-medication practices had become widely popular among people across countries. Considering its harmful and dangerous effects, the engagement of healthcare administrators and policymakers and the implementation of health education programs are essential to regulate and monitor self-medication practices (Zheng et al.).

In summary, the articles included in the topic are related to the transformative changes in the approach to health professions education due to the COVID-19 pandemic. Despite enormous challenges, students and educators continued engaging through the virtual learning environment. The use of digital technology is inevitable in future health professions education. Educational institutions need a high level of preparedness and establish digital infrastructure; design online or blended curricula; explore multi-modal approaches in online delivery and assessment; and conduct advanced-level research to integrate digital technology in health professions education.

## Author contributions

PS: Validation, Writing—original draft. HD: Validation, Writing—review and editing.
